# The Role of Parental Involvement in the Development of Prosocial Behavior in Young Children: An Evolutionary Model Among Colombian Families

**DOI:** 10.1007/s10578-024-01762-7

**Published:** 2024-10-29

**Authors:** M. Prada-Mateus, D. Obando, J. Sandoval-Reyes, M. A. Mejía-Lozano, J. Hill

**Affiliations:** 1 Depertment of Psychology and Behavioral Sciences, Universidad de La Sabana, Autopista Norte de Bogotá. Chía, Campus del Puente del Común, Km. 7, Cundinamarca, Colombia; 2 Department of Psychology, Manchester Metropolitan University, Manchester, UK

**Keywords:** Prosocial behavior, Parental involvement, Early childhood, PLS path modeling

## Abstract

Prosocial behavior is a relevant indicator of children’s socio-emotional development linked to decreased conduct and emotional problems. The present study aimed to identify cross-sectional direct effects of parental involvement on prosocial behavior in three-time assessments at ages 3, 5, and 7 years, to identify carryover effects of the study constructs, and to identify the evolution of these effects over time. A sample of 235 Colombian families participated at t0, 220 at t1, and 145 at t2 by completing self-reported questionnaires for prosocial behavior using the Strengths and Difficulties Questionnaire and the Alabama Parenting Questionnaire for parental involvement. Using PLS-SEM path modeling, we found that the contribution of parental involvement to prosocial behavior was significant in the three assessments. Carryover analyses indicated that initial levels of parental involvement and initial levels of prosocial behavior predict later levels. Using multigroup analysis, we tested significant changes in the path coefficients of direct effects, finding nonsignificant results. For carryover effects, we found changes in parental involvement between t0/t1 and t1/t2. Finally, t-test analyses were used to identify changes in the construct’s means over time, finding significant changes between parental involvement at t1 and t2. No mean differences were found for prosocial behavior. Results from this study highlight the relevance of parental involvement during childhood for maintaining children’s levels of prosocial behavior and reducing the risk of socio-emotional problems. Preventive approaches for these problems should include parents’ training on parental involvement from age 3.5 years or earlier.

## Introduction

Prosocial behavior is crucial to children’s socioemotional and interpersonal development and general well-being; it is a voluntary act intending to benefit and satisfy others’ needs for physical and emotional support [1]. This behavior facilitates proper development from childhood to adulthood [1, 2] as it is a protective factor for antisocial behaviors [3, 4]. In this line, different studies proposed that prosocial behavior reduces the risk of aggressive or undesirable behaviors since it mitigates the probability of its occurrence and promotes positive experiences and social adjustment [5–7]. Moreover, prosocial behavior is linked to interpersonal skills [8], positively influencing individual development, social exchanges, and enhancing social responsibility [9].

Prosocial behavior is settled during early childhood, between ages 18 and 24 months, and develops according to cultural, contextual, biological, dispositional, situational, and social factors [1, 10, 11]. Between middle childhood (ages 5 to 8 years), these factors and experiences are responsible for the variance of prosocial behavior, its maintenance, and its increase in adolescence and adulthood [8, 12].

Remarkably, family represents a relevant context for children’s prosocial behavior. Children acquire behaviors primarily by interacting with their parents in the family setting through parental modeling [13]. Regarding children’s prosocial behavior, modeling can occur when parents perform empathic and prosocial acts directly toward the child or in interactions with others, predisposing children’s future actions based on positive social exchanges [14]. For instance, prospective studies have provided evidence that positive parenting predicts increased levels of prosocial behavior: these studies include the exploration of parental positive reinforcement [15] and warmth in preschoolers [16], and parental warmth, sympathy [17], sensitivity [18] and empathy in children [19].

Two longitudinal studies in different countries, including Medellín, Colombia families, explored positive parenting dimensions and their effect on prosocial behavior [20, 21]. The study by Pastorelli found that children’s prosocial behavior at ages 9 and 10 increases mother–child positive interactions, but parenting did not predict prosocial behavior. However, Putnick’s later study found that positive discipline and parental acceptance were related to increased prosocial behavior in children. Other studies in Colombia have focused on describing and comparing the prevalence of prosocial behavior, finding that prosocial behavior prevalence is lower in this population compared to countries such as Argentina and Spain [22–24]. However, no studies were found on the relevance of parental involvement in prosocial behavior in children.

Parental involvement is a positive parenting dimension linked to positive behaviors since early childhood. This dimension comprises different positive parent–child interactions such as warmth, engagement, and responsiveness [25, 26], prompting children’s empathy through the understanding of the relevance of emotionally based social interactions [27, 28]. According to Piotrowska et al. [29], this dimension consists of “paying attention, being receptive and open to new ways of interacting with children, actively contributing to discussions and tasks, completing homework tasks or asking questions” (p. 150). Studies have explored the relationship between parental involvement and positive children’s outcomes, such as academic achievement [30, 31] and psychological adjustment [32].

Therefore, parental involvement may constitute a protective factor for children’s mental health in the Colombian context in which several risk factors are presented, including poverty, exposure to violence and war-related experiences, low parental educational levels, and negative parenting [33]. Also, there is evidence regarding the use of punitive practices among Colombian parents since early childhood [34] and reports of a high prevalence of conduct disorders in Colombian children [35].

**Fig. 1:**
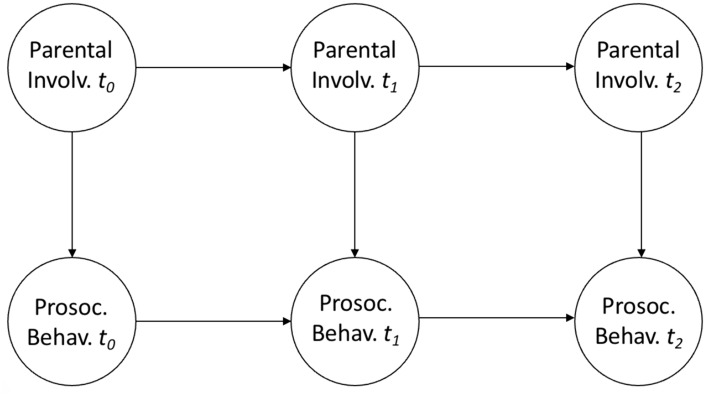
We hypothesized evolution model for parental involvement and prosocial behavior direct and carryover effects

The study objective was to identify the evolution of direct effects of parental involvement on children’s prosocial behavior and the carryover effects of these constructs (Fig. 1). From a longitudinal perspective, three-time assessments at children’s 3.5 years (t0), 5 years (t1), and 7 years (t2) were conducted. Data was analyzed using Partial Least Squares Structural Equation Modelling (PLS-SEM) evolution model for panel data [36] to test four hypotheses: (1) parental involvement contributes to increased levels of prosocial behavior at three times, (2) initial levels of parental involvement predicts later levels of this parental dimension, and initial levels of prosocial behavior predict later levels of this behavior; (3) the effect of parental involvement on prosocial behavior will become stronger over time; (3b) the carryover effects will become stronger over time for both parental involvement and prosocial behavior; (4) the mean levels of parental involvement and prosocial behavior will increase over time.

## Methods

### Participants

The sample of the present research is part of a broader prospective study called the La Sabana Parent–child Study, which aims to examine the association between parenting dimensions and children’s socioemotional development [37]. The three assessments were completed with families from three Colombian regions (Pacific, Central and Caribbean) who were invited through Facebook’s groups: at t0 235 families with children with a mean age of 3.31 (SD = 0.47) participated after signing the informed consent, in t1 220 (97%) families from the initial sample (children’s mean age = 4.9, SD = 0.42); and in t2 150 families (63.5% of the initial sample, children’s mean age = 7.2, SD = 0.39). Assessments in the three times had a similar percentage of boys and girls (49% girls), and the percentage of families classified as low-income families was between 45 and 47%.

### Instruments

*The Strengths and Difficulties Questionnaire (SDQ; *[38]*)*: This questionnaire measures psychological adjustment in children aged 3 to 16 years old through 25 items divided into five scales: emotional symptoms, conduct problems, hyperactivity/inattention, peer problems, and prosocial behavior (e.g., “Takes into account other people’s feelings” and “Frequently shares knick-knacks, toys, pencils, etc. with other children.”). Answer options are presented on a three-point scale (0 = “not true,” 1 = “somewhat true,” and 2 = “certainly true”). The level of prosocial behavior is calculated by adding the five items of the scale (1, 4, 9, 17, and 20). The number of items at each time assessment varied when running the PLS-SEM analysis. Internal reliability was acceptable at the three-time assessments [39]: at t0, items 1, 17, and 20 were retained (α = 0.64),at t1, items 1, 9, 17, and 20 (α = 0.63); and t2 all items (α = 0.68) (Appendix 1).

*Alabama Parenting Questionnaire (APQ; *[40]*):* The APQ comprises 42 items to assess positive and negative parenting dimensions: inconsistent discipline, monitoring, corporal punishment, positive reinforcement, and parental involvement. For t0 and t1, the APQ preschooler version was used (APQ-Pr, [41]), and for t2 the APQ. Items for parental involvement (variable of relevance in this study) include questions such as “You have a friendly talk with your child” and “You join your child’s favorite activities”. At t0 and t1, APQ-Pr items 1, 4, 6, 8, 9, and 16 were retained (α = 0.73 and 0.69, respectively), and at t2, items 1, 4, 7, 9, 11, 14, 20, 23 and 26 (α = 0.69) (Appendix 2).

### Procedure

The assessment at t0 was completed in the families’ households, where parents signed the informed consent and completed questionaries. Informers mainly were mothers (93.6%), followed by fathers (4.6%) and grandmothers (1.7%). Data at t1 and t2 was gathered using digital versions of the questionnaires. After each assessment, families received incentives for the children (books, toys, or didactic material) and parents (money). The Universidad de La Sabana Ethics and Research Committee approved the study through minute 102 on May 3rd, 2017.

### Data Analysis

Using the PLS-SEM evolution model for panel data, we first explored the association between parental involvement and prosocial behavior at three-time assessments (direct effects). Then, we studied the association between initial and later levels of parental involvement and prosocial behavior (carryover effects). Additionally, we explored whether significant changes between direct associations and carryover effects were observed over time, including mean differences analyses. This type of analysis helps predict constructs in evolutionary models, dealing with complex models due to the number of constructs measured at separate times and dealing with longitudinal studies with small sample sizes [36].

The measurement model met the quality criteria of convergent and discriminant validity and composite reliability. Following previous literature recommendations, all the factor loadings were greater than 0.5. Fornell-Larcker Criterion and HTMT criteria supported the model’s discriminant validity [42–44]. This process guarantees that the measurement model fulfills the standards for PLS modeling. Analyses were conducted using Smart PLS 4 [45].

## Results

**Fig. 2:**
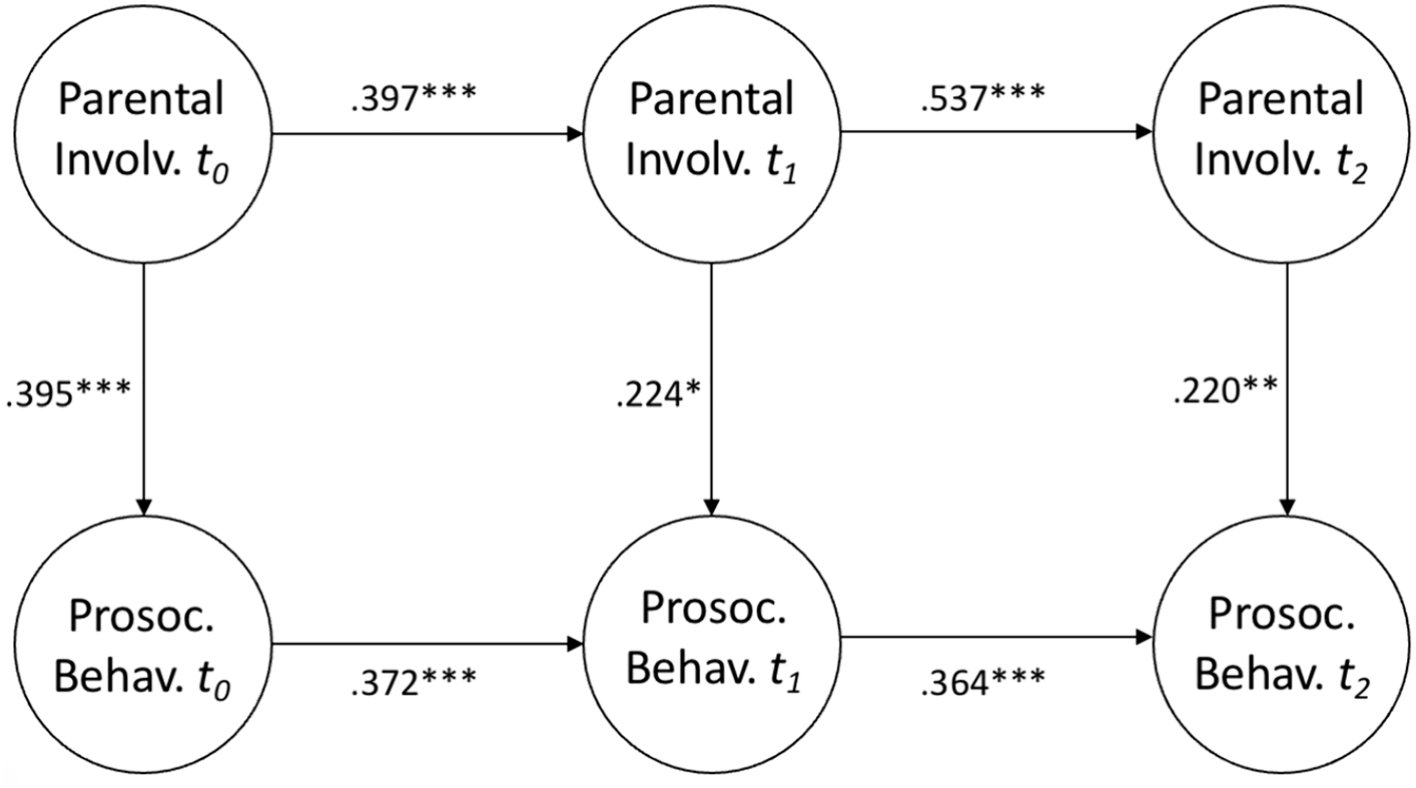
Evolution model for panel data for parental involvement and prosocial behavior direct and carryover effects. ***p < .005; **p < .010; *p < .050

Results regarding the direct and carryover effects of the study variables are presented in Fig. 2 and Table 1.

**Table 1:** Significance of the direct and carryover effects of parenting involvement and prosocial behavior at three-time assessment

Type	Time	Effect	Path coefficient	t	p	Significance
Direct Effects	t0	Inv t0 → Pro t0	.395	5.335	.000	Yes
	t1	Inv t1 → Pro t1	.224	2.408	.016	Yes
	t2	Inv t2 → Pro t2	.220	2.745	.006	Yes
Carryover effects	t0/t1	Inv t0 → Inv t1	.397	4.696	.000	Yes
	t1/t2	Inv t1 → Inv t2	.537	8.283	.000	Yes
	t0/t1	Pro t0 → Pro t1	.372	4.470	.000	Yes
	t1/t2	Pro t1 → Pro t2	.364	4.533	.000	Yes

*Inv* parental involvement, *Pro* prosocial behavior

Figure 2 and Table 1 indicate that the path coefficients were significant for direct and carryover effects. Parental involvement predicts children’s prosocial behavior at the three-time assessments, being stronger in t0 (age 3.5 years) compared to t1 and t2. Carryover effects were significant for parental involvement and prosocial behavior, being stronger for parental involvement between t1 and t2.

Multigroup analyses were conducted to identify whether changes in the association between the study variables (direct effects) over time and changes in the associations between constructs from one time to another (carryover effects) were significant (Table 2).

**Table 2:** Significance of the changes in path coefficients for direct and carryover effects

Type	Time	Effect	Path Coeff	Size of the change	Bias corrected CI	Comparison of path coefficient t + 1 with CI t and path coefficient t with CI t + 1	Path coefficient t + 1 inside CI t? Path coefficient t inside CI t + 1?	Sign. change
Direct Effects	t0	Inv t0 → Pro t0	.395	-.171	(.221; .515)	.221 < .224 < .515	Yes	No
	t1	Inv t1 → Pro t1	.224		(.037; .401)	.037 < .395 < .401	Yes	
	t1	Inv t1 → Pro t1	.224	-.004	(.037; .401)	.037 < .220 < .401	Yes	No
	t2	Inv t2 → Pro t2	.220		(.047; .361)	.047 < .224 < .361	Yes	
Carryover effects	t0/t1	Inv t0 → Inv t1	.397	.140	(.187; .534)	.187 < .537	No	Yes
	t1/t2	Inv t1 → Inv t2	.537		(.386; .642)	.386 < .397 < .642	Yes	
	t0/t1	Pro t0 → Pro t1	.372	-.008	(.182; .515)	.182 < .364 < .515	Yes	No
	t1/t2	Pro t1 → Pro t2	.364		(.168; .500)	.168 < .372 < .500	Yes	

*Inv* parental involvement, *Pro* prosocial behavior, *Coeff*. Coefficient, *Sign*. significance

Results in Table 2 show no significant changes between the three assessments regarding the direct effects of parental involvement and prosocial behavior. For carryover effects, there was a significant change between parental involvement at t0/t1 and t1/t2. No changes were observed in prosocial behavior over time.

A T-test analysis was conducted to identify significant changes in mean parental involvement and prosocial behavior levels at the three assessments. The results are presented in Table 3.

**Table 3:** Results of the test significance of the changes in the constructs’ levels

Construct	Time	No. of Pairs	M	SD	Mean Difference	t	p	Sign
Parental involvement	t0 to	150	4.37	0.54				
	t1	150	4.41	0.47	-0.04	-0.885	.377	No
	t1 to	150	4.41	0.47				
	t2	150	4.21	0.49	0.21	5.453	< .001	Yes
Prosocial behavior	t0 to	150	1.69	0.39				
	t1	150	1.70	0.33	-0.01	-0.327	.744	No
	t1 to	150	1.70	0.33				
	t2	150	1.73	0.30	-0.03	-0.730	.466	No

*Sign*. significance

Data in Table 3 indicates a significant difference between parental involvement means at t1 (4.37) and t2 (4.41). No differences were found between prosocial behavior means.

## Discussion

The present study aimed to identify the evolution of the direct effects between parental involvement and prosocial behavior and the carryover effects between the initial and later levels of the study variables. Furthermore, the study explored whether changes over time for both direct and carryover effects were significant in contributing to the identification of mechanisms involved in the maintenance and increase of prosocial behavior in young children, which is considered a protective factor for behavioral and emotional problems over time [8, 46].

Results confirmed hypothesis one, in which parental involvement was predicted to contribute to prosocial behavior in children aged 3.5, 5, and 7. This result supports previous findings (e.g., [12, 16, 17, 47]), providing additional evidence about the relevance of positive parenting on children’s positive behavior regardless of sociodemographic and cultural characteristics across countries. For the present study, direct effects were maintained over time, informing a stable pattern of the association between variables across the different age groups. Therefore, interventions promoting parental involvement could positively impact children’s prosocial behavior across various developmental stages by prompting children’s prosocial exchanges and identifying others’ emotional states [28, 48].

It is relevant to highlight that the contribution of parental involvement to prosocial behavior was stronger in children aged 3.5 years compared to ages 5 and 7 years. Younger children are more sensitive to parental influences than older children, and their behavior influences, to a greater extent, the acquisition of specific repertories, such as positive social interactions [18]. While children grow older, they are exposed to additional influences beyond parental interactions that may play a relevant role in shaping social interactions [49]. Intervention and preventive approaches must adapt programs regarding children’s ages considering developmental characteristics.

Hypothesis two stated that initial levels of the constructs predict later levels. We found that parental involvement at age 3.5 predicted parental involvement at age 5, which predicted it at age 7. It was observed that the association between parental involvement at age 5 and age 7 was stronger compared to the association between age 3.5 and 5 years. This finding might be related to the fact that parenting interactions at early stages could vary from those observed when children get older. Variations could be associated with children’s characteristics, parental circumstances, previous experiences, social support, and cultural factors [50].

Prosocial behavior carryover effects were significant and similar between ages. Our results are consistent with other studies, such as those by Eisenberg et al. [1, 10], indicating that early childhood prosocial behaviors can be identified. Moreover, early prosocial behavior anticipates future levels of this protective factor. Therefore, early preventive strategies aiming at reducing the risk of children’s mental health problems must include parents’ training in positive and involved interactions and fostering the child’s repertory of prosocial interactions for persisting effects throughout developmental stages [51, 52].

Hypothesis three was not corroborated in the present study: multigroup analyses indicated that the direct effect of parental involvement on prosocial behavior did not change over time, as proposed. This result implies that the association between the variables at different ages remains similar, suggesting that children’s age does not influence how these variables relate. Again, this points out the relevance of parental involvement in prosocial behavior regardless of the children’s age. The hypothesis regarding carryover effects changes (3b) was ratified for parental involvement: we found a significant change between t0/t1 and t1/t2. This finding is linked and complementary to hypothesis two, confirming that carryover effects for parental involvement become stronger over time. Temporal changes in parental involvement align with our previous analysis, in which we stated that parent–child interactions must adapt to the child’s socio-emotional needs at each period. Understanding this is relevant for tailored strategies with parents to promote involved behaviors corresponding to children’s age. On the contrary, prosocial behavior had no significant changes over time; this may be related to stable individual tendencies influencing social behavior [53]. However, as we have evidenced, these tendencies are susceptible to external influences, such as parenting, to be enhanced and maintained [54].

Finally, hypothesis four stated that parental involvement and prosocial behavior levels will increase over time. Our results contradicted this hypothesis, showing that the mean for parental involvement at t2 decreased significantly from t1. As children grow older, parents engage in other activities within the work and social contexts, and children get more independence as they expand their social and academic activities [55], leading to less time to be involved together. From a preventive perspective, it is relevant to promote parents’ identification of new scenarios and activities to interact with their children based on warm, engaged, and responsive interactions. No mean differences were found regarding children’s prosocial behaviors, which aligns with the previously discussed stability of social interaction tendencies.

In conclusion, enhancing parental involvement since early childhood promotes prosocial behavior, leading to a reduced risk for mental health problems. This study is informative about the role of parental involvement beyond children’s academic performance as it is relevant for promoting empathy and altruism. This link is maintained over time even though parent–child-involved interaction behaviors may vary from one age to another. As we found stability over time in children’s prosocial behavior, preventive approaches must focus on early promoting this behavior through parental involvement, increasing the opportunity for internalization and recognition of empathic and prosocial interactions. In addition, the stronger effect of parental involvement on prosocial behavior at age 3.5 suggests that early interventions may have a more significant impact on shaping children’s prosocial behaviors.

The present study’s strengths include the following: First, this is one of the first longitudinal studies exploring the role of parental involvement on prosocial behavior in young Latin American children. Second, the sample characteristics represent the Colombian population regarding sex, SES, cultural diversity, and participants’ ethnicity. Third, we used measures that previously showed good internal consistency among samples with similar characteristics. Fourth, our results have provided the basis for designing preventive strategies delivered to Colombian families, including those participating in this study. Strategies include a brief program of group sessions to promote parental involvement and reinforce children’s prosocial behaviors and digital material such as videos from clinical psychologists and infographics regarding the study results and strategies to promote both behaviors.

Some study limitations include using self-reported measures, where participants’ subjectivity and social desirability do not restrict information. The internal reliability of the scales was acceptable, and the SDQ and APQ measurements have not been previously validated with Colombian samples. There was a low retention rate for the t2 assessment. Thus, further research may include additional measures for assessing prosocial behavior to reduce common method bias, such as assessing prosocial behavior from children’s reports. Moreover, future research may consider assessing different types of prosocial behavior observed during childhood [12].

## Summary

The present longitudinal study explored the relationship between parental involvement and children’s prosocial behavior during early developmental stages. Specifically, it aimed to identify the evolution of direct and carryover effects of parental involvement at ages 3.5, 5, and 7 years, and how these effects contributed to the development and maintenance of prosocial behaviors in children, a protective factor against behavioral and emotional problems.

The results provided evidence that parental involvement positively influences prosocial behavior, with stronger effects observed at earlier ages (3.5 years), highlighting the importance of early intervention. As children grow older, external influences have a more significant role in their social interactions, which suggests the need for tailored strategies for different developmental stages. The study found consistent carryover effects of prosocial behavior across ages, emphasizing the stability of prosocial tendencies over time. However, contrary to our hypothesis, parental involvement levels decreased as children aged, likely due to parents’ changing circumstances and children’s increasing independence.

## Data Availability

Datasets can be accessed under request to the corresponding author.

## Appendix 1: Table of items of SDQ for prosocial participation

**Table:**

Time	Items
t0	Item 1: Considerate of other people’s feelings Item 17: Kind to younger children Item 20: Often volunteers to help others (parents, teachers, other children)
t1	Item 1: Considerate of other people’s feelings Item 9: Helpful if someone is hurt, upset, or feeling ill Item 17: Kind to younger children Item 20: Often volunteers to help others (parents, teachers, other children)
t2	Item 1: Considerate of other people’s feelings Item 4: Shares readily with other children Item 9: Helpful if someone is hurt, upset, or feeling ill Item 17: Kind to younger children Item 20: Often volunteers to help others (parents, teachers, other children)

## Appendix 2: Table of items of APQ-PR and APQ for prosocial participation

**Table:**

Time	Items
t0 APQ-Pr	Item 1: You have a Friendly talk with your child Item 4: You volunteer to help with special activities that your child is involved in Item 6: You play games or do other fun things with your child Item 8: You ask your child about his/her day in school Item 9: You help your child with his/her homework Item 16: You talk to your child about his/her friends
t1 APQ-Pr	Item 1: You have a Friendly talk with your child Item 4: You volunteer to help with special activities that your child is involved in Item 6: You play games or do other fun things with your child Item 8: You ask your child about his/her day in school Item 9: You help your child with his/her homework Item 16: You talk to your child about his/her friends
t2 APQ	Item 1: You have a Friendly talk with your child Item 4: You volunteer to help with special activities that your child is involved in such as sports boy/girl scouts, church, youth groups Item 7: You play games or do other fun things with your child Item 9: You ask your child about his/her day in school Item 11: You help your child with his/her homework Item 14: You ask your child what his/her plans are for the coming day Item 20: You talk with your child about his/her friends Item 23: Your child helps plan family activities Item 26: You attend PTA meetings, parent/teacher conferences, or other meetings at your child’s school
